# Genome Report: Whole Genome Sequence and Annotation of the Parasitoid Jewel Wasp *Nasonia giraulti* Laboratory Strain RV2X[u]

**DOI:** 10.1534/g3.120.401200

**Published:** 2020-06-22

**Authors:** Xiaozhu Wang, Yogeshwar D. Kelkar, Xiao Xiong, Ellen O. Martinson, Jeremy Lynch, Chao Zhang, John H. Werren, Xu Wang

**Affiliations:** * Department of Pathobiology, Auburn University, AL 36849,; ^†^ Department of Biology, University of Rochester, NY 14627,; ^‡^ Translational Medical Center for Stem Cell Therapy and Institute for Regenerative Medicine, Shanghai East Hospital, Shanghai Key Laboratory of Signaling and Disease Research, School of Life Sciences and Technology, Tongji University, China,; ^§^ Department of Biology, University of New Mexico, Albuquerque, NM 87131,; ^**^ Department of Biological Science, University of Illinois at Chicago, IL 60607,; ^††^ HudsonAlpha Institute for Biotechnology, Huntsville, AL 35806,; ^‡‡^ Alabama Agricultural Experiment Station, Auburn, AL 36849, and; ^§§^ Department of Entomology and Plant Pathology, Auburn University, AL 36849

**Keywords:** *Nasonia*, parasitoid wasp, linked-reads technology, whole-genome sequencing, genome assembly

## Abstract

Jewel wasps in the genus of *Nasonia* are parasitoids with haplodiploidy sex determination, rapid development and are easy to culture in the laboratory. They are excellent models for insect genetics, genomics, epigenetics, development, and evolution. *Nasonia vitripennis* (*Nv*) and *N. giraulti* (*Ng*) are closely-related species that can be intercrossed, particularly after removal of the intracellular bacterium *Wolbachia*, which serve as a powerful tool to map and positionally clone morphological, behavioral, expression and methylation phenotypes. The *Nv* reference genome was assembled using Sanger, PacBio and Nanopore approaches and annotated with extensive RNA-seq data. In contrast, *Ng* genome is only available through low coverage resequencing. Therefore, *de novo Ng* assembly is in urgent need to advance this system. In this study, we report a high-quality *Ng* assembly using 10X Genomics linked-reads with 670X sequencing depth. The current assembly has a genome size of 259,040,977 bp in 3,160 scaffolds with 38.05% G-C and a 98.6% BUSCO completeness score. 97% of the RNA reads are perfectly aligned to the genome, indicating high quality in contiguity and completeness. A total of 14,777 genes are annotated in the *Ng* genome, and 72% of the annotated genes have a one-to-one ortholog in the *Nv* genome. We reported 5 million *Ng-Nv* SNPs which will facility mapping and population genomic studies in *Nasonia*. In addition, 42 *Ng*-specific genes were identified by comparing with *Nv* genome and annotation. This is the first *de novo* assembly for this important species in the *Nasonia* model system, providing a useful new genomic toolkit.

*Nasonia* wasps have a parasitoid lifestyle, where females inject venom into fly pupal hosts and then deposit eggs onto the fly puparium. The venom induces developmental arrest and changes in host gene expression and metabolism (Danneels *et al.* 2010; Mrinalini *et al.* 2015; Martinson *et al.* 2014), with the feeding wasp larvae eventually killing the host. There are four species in the genus including *N. vitripennis* (*Nv*), *N. giraulti* (*Ng*), *N. oneida* (*No*) and *N. longicornis* (*Nl*) (Darling and Werren 1990; Raychoudhury *et al.* 2010; Whiting 1967). *Nv* was the first and only species described in this genus for a long period of time and has a worldwide distribution (Whiting 1967). *Ng*, *Nl* and *No* are closely-related to *Nv* and have a more restricted North American distribution (Figure 1), where they parasitize blowfly pupae in birds’ nests (Darling and Werren 1990; Raychoudhury *et al.* 2010). The *Nasonia* sex determination mechanism is haplodiploidy, which is shared among Hymenoptera (Lynch 2015; Werren and Loehlin 2009; Whiting 1967). Reproductive incompatibility due to *Wolbachia*-induced cytoplasmic incompatibility occurs in *Nasonia*, except for *Ng*/*No* (Breeuwer and Werren 1990; Bordenstein *et al.* 2001). However, interspecies interspecific hybrids of *Nasonia* are readily generated after antibiotic curing the wasp of *Wolbachia* (Werren and Loehlin 2009). In addition, there is a rapidly expanding genetic toolkit for *Nasonia* (Lynch 2015), including recent advances in germline transformation techniques (Chaverra-Rodriguez *et al.* 2020).

**Figure 1:**
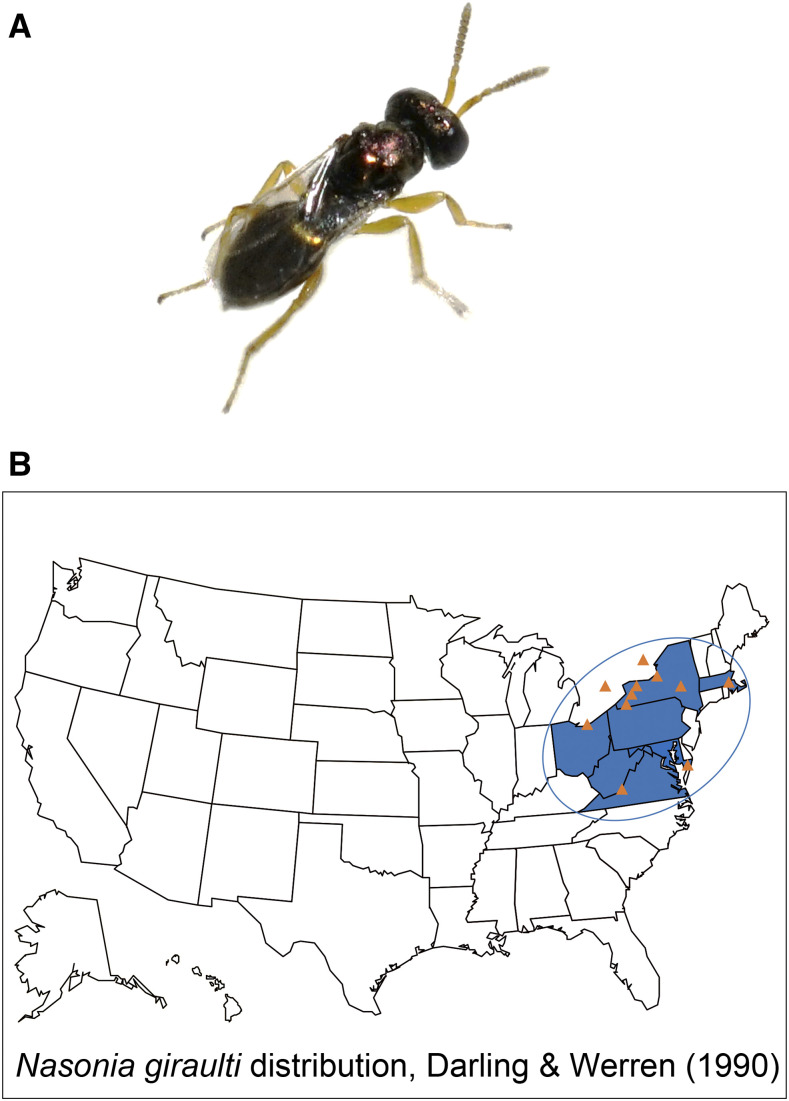
Image of *N. giraulti* and its geographic distribution in the North America based on Darling & Werren (1990).

*Nasonia* has been a good model for insect research (Werren and Loehlin 2009; Lynch 2015; Whiting 1967; Beukeboom and Desplan 2003). Whole-genome sequencing efforts have been made in *Nv*, *Ng* and *Nl* (Werren *et al.* 2010). The *Nv* genome was sequenced with 6X coverage Sanger sequencing to generate a *de novo* assembly, whereas *Ng* and *Nl* genomes were sequenced with 1X coverage supplemented with short-read sequences, and aligned to the *Nv* assembly for reference-based genomes (Werren *et al.* 2010). Plenty of datasets have been published for *Nv* genome and transcriptomes after its reference genome was available. Crosses between *Nv* and *Ng* have been extremely successful for mapping and positional cloning of genes involved in species differences (Werren and Loehlin 2009; Niehuis *et al.* 2013), in some cases using chromosomal regions of *Ng* introgressed into an *Nv* background (Hoedjes *et al.* 2014). Comparative genomics between *Ng* and *Nv* is informative to investigate many aspects in *Nasonia* biology, such as behavior (Raychoudhury *et al.* 2010), development (Loehlin and Werren 2012), pheromones (Niehuis *et al.* 2013), sex determination (Verhulst *et al.* 2010), gene expression (Wang *et al.* 2015, 2016; Rago *et al.* 2020), venom evolution (Martinson *et al.* 2017) and regulation by DNA methylation (Beeler *et al.* 2014; Pegoraro *et al.* 2016; Wang *et al.* 2013). Therefore, a well-assembled reference genome of *Ng* will advance utility of the system by the research community. In this study, we generated a high-quality reference genome assembly for *N. giraulti*, which will provide essential new genomic tools for *Nasonia* research.

## Materials and Methods

### DNA extraction, library preparation, and sequencing

DNA was extracted from 24-hour male adults of the *N. giraulti* RV2X[u] strain. High molecular weight (HMW) genomic DNA (gDNA) was isolated using MagAttract HMW DNA Mini Kit (Qiagen, MD). The quality of extracted gDNA was examined on a Qubit 3.0 Fluorometer (Thermo Fisher Scientific, USA). The size distribution of the extracted gDNA was accessed using the genomic DNA kit on Agilent TapeStation 4200 (Agilent technologies, CA).

A 10X Genomic library was prepared with the Chromium Genome Reagent Kits v2 on the 10X Chromium Controller (10X Genomics Inc., CA). In brief, HMW gDNA was diluted from original concentrations to ∼0.9 ng/µl with EB buffer. The diluted denatured gDNA, sample master mix and gel beads were loaded to the genomic chip, and then ran on 10X Chromium Controller to create Gel Bead-In-EMulsions (GEMs). After the run, the obtained GEMs were used for the subsequent incubation and cleanup. Chromium i7 Sample Index was used as the library barcode. Quality control of post library construction was accessed with Qubit 3.0 Fluorometer and Agilent TapeStation 4200. The prepared 10X genomic library was sequenced on a HiSeq X sequencer at the Genomic Services Lab at the HudsonAlpha Institute for Biotechnology. An Illumina short-read resequencing library (300 bp insert size) was made from genomic DNA samples extracted from six *N. giraulti* adult males (whole body), using TruSeq DNA Sample Prep Kit. Approximately 50X paired-end sequencing was done using Illumina HiSeq 2000 platform.

### Total RNA extraction, library preparation, and sequencing of developmental stage samples

Male and female *N. giraulti* RV2X(u) strain samples were collected at five developmental stages: 0-10hr early embryo, 14-24hr late embryo, 44-54hr larva, yellow pupa and 1-day adult. *Sarcophaga bullata* pupae were inserted into foam plugs, with only anterior available for oviposition. To obtain the male samples, two host pupae were provided to two virgin female wasps, allowing host feeding for 48 hr. These unmated females lay unfertilized eggs and produce all-male progeny. For female sample collection, mated females will produce more than 90% daughters under the experimental conditions, allowing the expression quantification of mostly female progeny for embryo and larva stages. Six individuals were pooled per stage, except early embryos for which 40 individuals were pooled due to the small size. All samples were homogenized in 1 mL TRIzol and stored at -80C freezer. Total RNA extractions, quantification, library preparation and sequencing protocol were previously described (Martinson *et al.* 2017).

### Genome assembly and assessment

The raw sequencing reads from both 10X library and Illumina resequencing library were checked for sequencing quality by FastQC v11.5 (Andrews 2010) before used for genome assembly. The genome assembly strategy of *N. giraulti* includes the constructions of three draft *de novo* assemblies using different assemblers and a final step to reconcile three draft assemblies into a final high-quality assembly. The first *de novo* assembly of *N. giraulti* genome was performed with the Supernova 2.0 assembler (Weisenfeld *et al.* 2017) using linked reads from 10X Genomics library. To achieve the best *de novo* assembly result, we examined a grid of barcode subsampling percentage parameters and the maximum number of input reads including no barcode subsampling with all linked reads. A second *de novo* assembly was conducted by MEGAHIT v1.2.9 (Li *et al.* 2015). The 10X linked reads were transferred to regular paired-end Illumina sequencing reads by trimming the barcode sequences and potential adaptor sequences with Trimmomatic v0.38 (Bolger *et al.* 2014). All trimmed sequencing reads were used for the second *de novo* assembly using MEGAHIT v1.2.9 (Li *et al.* 2015) with all default parameter settings. In addition, a third *de novo* assembly (ngirB_goodCOV) was generated by velvet v1.2.10 (Zerbino and Birney 2008) using sequencing reads from the Illumina short-read resequencing library.

A final high-quality assembly was generated by merging these three draft assemblies using an assembly reconciliation tool Metassembler v1.5 (Wences and Schatz 2015). All reverse complementary scaffolds with same length, coverage, A/T/C/G counts, as well as the duplicated scaffolds identified by self-BLAT version 35 (Kent 2002) were removed from the final assembly. In addition, potential contaminating bacterial scaffolds were checked and removed from the assembly, using a combination of methods mentioned in our previous publications (Wang *et al.* 2019; Wheeler *et al.* 2013; Ferguson *et al.* 2020). To estimate the contiguity and completeness of our genome assembly, three evaluation pipelines were performed: (1) genome sequencing reads were aligned to our assembly with BWA-MEM aligner version 0.7.17 (Bernt *et al.* 2013); (2) transcriptomic data of different developmental stages and sexes were mapped to the current assembly using Tophat v2.1.1 (Trapnell *et al.* 2009); (3) The BUSCO (Seppey *et al.* 2019) score of our genome assembly was calculated by aligning to arthropoda_odb9 with a total of 1,066 orthologs.

### Genome annotation

The annotation of the *N. giraulti* genome was performed using MAKER version 2.31.9 (Cantarel *et al.* 2008) based on the following pipeline: (1) A custom *N. giraulti* repeat database constructed with RepeatModeler v.1.08 using the default parameter settings, with low complexity repeat regions soft-masked by MAKER; (2) A *de novo* assembly of the *N. giraulti* transcriptomes by Trinity v 2.4.0 (Haas *et al.* 2013) and pre-aligned transcripts annotated by Cufflinks v2.2.1 (Trapnell *et al.* 2012).For gene annotation, *ab initio* gene prediction algorithms were trained to predict gene models using protein and transcriptome evidences by EST2GENOME and PROTEIN2GENOME in MAKER. After filtered based on gene length and quality, the predicted genes were then used to train both the SNAP and the AUGUSTUS gene predictors. The results were fed to MAKER to repeat this procedure for another round, to generate the final predicted genes in *N. giraulti* genome. Default parameters were used except where otherwise noted.

### Comparitive ananlysis between N. giraulti and N. vitripennis genomes

To compare the genome structure between *N. giraulti* and *N. vitripennis* genomes, we conducted whole-genome alignment of our *Ng* assembly and the recent *Nv* genome assembly of (Dalla Benetta *et al.* 2020) using NUCmer in the MUMmer v4.0 program suite with default p parameter settings (Kurtz *et al.* 2004). The pairwise alignments (match length longer than 500bp) between *Ng* scaffolds and *Nv* chromosomes were visualized using Mummerplot (Kurtz *et al.* 2004).

To identify the candidate *Ng* specific genes, genes with no assigned orthogroup between *N. giraulti* and *N. vitripennis* were generated using OrthoFinder v2.2.7 (Emms and Kelly 2019). The *Ng* genes identified to have no assigned orthogroup with *Nv* were potential candidates for *Ng* specific genes. To ensure the absence of these candidates in *Nv* genome, protein sequences of these candidate *Ng*-specific genes were BLASTed to two *Nv* genome assemblies, including the *Nv* reference genome assembly (GCA_000002325.2) (Werren *et al.* 2010) and the newly released *Nv* PSR1.1 genome assembly using PacBio and Nanopore platforms (GCA_009193385.1) (Dalla Benetta *et al.* 2020) with an E-value cutoff of 1E-5 and protein length larger than 30. Genes with no BLAST hit to the two *Nv* genome assemblies were then aligned to the annotated *Ng* transcripts. The annotated *Ng* transcripts were generated with available *Ng* RNA-Seq data from different developmental stages and sexes (Embryo stage of 0-10 hr, 10-24 hr, 24-36 hr, female and male pupa and adult) using Cufflinks (Trapnell *et al.* 2012). Genes with support from annotated transcripts were kept as *Ng*-specific candidates. The protein sequences of these genes were aligned to the *Nv* PSR1.1 and *Trichomalopsis sarcophagae* assemblies using tBLASTn with an E-value cutoff of 1E-5. The final genes were annotated using both Blast2GO and KofamKOALA with an E-value cutoff of 1E-4.

### Phylogenomic analysis

We conducted a phylogenomic analysis using our assembled *N. giraulti* genome and 8 other sequenced insect genomes, including the fruit fly *Drosophila melanogaster* (GCA_000001215.4) (Adams *et al.* 2000), pea aphid *Acyrthosiphon pisum* (GCA_005508785.1) (International Aphid Genomics Consortium 2010), honey bee *Apis mellifera* (GCA_003254395.2) (Honeybee Genome Sequencing Consortium 2006), water flea *Daphnia pulex* (GCA_000187875.1) (Colbourne *et al.* 2011), human lice *Pediculus humanus* (GCA_000006295.1) (Kirkness *et al.* 2010), mosquito *Anopheles gambiae* (GCA_000005575.1) (Lawniczak *et al.* 2010), silk moth *Bombyx mori* (GCA_000151625.1) (Xia *et al.* 2004), and jewel wasp *Nasonia vitripennis* (GCA_000002325.2) (Werren *et al.* 2010). Homologous genes among these 9 genomes were identified using OrthoFinder (Emms and Kelly 2019, 2015) with default settings. The protein sequences of the core single-copy genes shared in all 9 genomes were aligned with MAFFT v7.407 (Katoh and Standley 2014). ProtTest 3 (Darriba *et al.* 2011) was used to evaluate The best-fit model of protein evolution. The Maximum Likelihood (ML) phylogenetic tree of the concatenated protein sequence was inferred by using RAxML v8.2 (Stamatakis 2014) with the VT protein model (best fit model identified by ProtTest 3) and 1,000 rapid bootstrap replicates.

### Data availability

The *Ng* genome assembly is available in GenBank with accession number QLYP00000000. Raw sequencing data are available in the NCBI Sequence Read Archive under the accession number PRJNA476699. Supplemental material available at figshare: https://doi.org/10.25387/g3.12433559.

## Results and discussion

### Genome assembly and assessment

Supernova 2.0 assembler (Weisenfeld *et al.* 2017) was used for the *Ng* genomic assembly with the barcode subsampling strategy. The best Supernova assembly has a contig N50 of 36.14 Kb and a scaffold N50 of 400.25 Kb, which was obtained by using 20% barcode subsampling of 140 million input reads. Interestingly, using all available reads with no barcode subsampling provided the worst assembly result. This can be caused by the overkill of reads coverage (>600X), which might lead to fragmented assembly due to the presence of sequencing errors. The draft *de novo* assembly was found to contain some artifacts, which was also reported for this assembler in a recent study (Helmkampf *et al.* 2019). We removed all the identical or nearly identical scaffolds as well as reverse complementary scaffolds prior to subsequent analyses. All these three *de novo* assemblies generated from different algorithms were further reconciled using an assembly reconciliation tool Metassembler (Wences and Schatz 2015). To identify the mitochondrial scaffold, we aligned the final assembly to the previously assembled mitochondrial genome of *N. giraulti*. Scaffolds with high identity (>90%) and high coverage (>16,000X) were assigned as mitochondrial scaffolds (Supplemental Figure S1).

The detailed genome statistics of our final assembly of *N. giraulti* and all other available wasp genomes, including previous assembled genomes are listed in Table 1. The final genome assembly of *N. giraulti* is a total of 259,040,977 bp in 3,160 scaffolds. The contig N50 is 34,917 bp and the scaffold N50 is 545,346 bp, respectively. The previous *Ng* assembly was based on 1X Sanger and 10X Illumina short-read alignments to an earlier *Nv* assembly (Werren *et al.* 2010). Comparing to reference-assisted *Ng* assembly, our *de novo* assembly was significantly improved in contig level with much lower number of contigs and larger contig N50. The gap percentage is only 1.5% of the whole assembly, which surpasses most of the previous *Nasonia* genome assemblies. Although the scaffold N50 of the whole *Ng* genome is ∼545 kb, the scaffold N50 of the protein coding gene-contained scaffolds (a total of 1,393 scaffolds) is 664.6 Kb, indicating the high quality of our current assembly in the genic regions.

**Table 1:** Statistics of the N. giraulti genome assembly compared to other wasp species

Genome assembly	Ngir_v5	Ngir_1.0	Nvit_2.1	Nlon_1.0	Tsac_v1
**Species**	*N. giraulti*	*N. giraulti*	*N. vitripennis*	*N. longicornis*	*T. sarcophagae*
**No. of scaffolds**	3,160	4,912	6,098	5,214	4,0891
**No. of contigs**	14,039	373,227	25,484	385,077	57,930
**Scaffold length (bp)**	259,040,977	283,606,953	295,780,872	285,726,340	236,484,274
**Contig length (bp)**	255,292,562	178,561,037	238,616,307	181,397,296	235,211,350
**Gap percentage**	1.5%	37.0%	19.3%	36.5%	0.5%
**Scaffold N50 (bp)**	545,346	759,431	897,131	758,407	22,350
**Contig N50 (bp)**	34,917	1,973	18,840	1,877	9,957
**Scaffold N90 (bp)**	46,391	62,470	46,455	59,334	2,779
**Contig N90 (bp)**	9,262	163	4,180	162	1,943
**Scaffold maximum length (bp)**	6,445,087	9,412,112	33,571,687	9,412,414	350,161
**Contig maximum length (bp)**	385,696	35,702	226,699	39,258	140,646
**Percentage of scaffold > 50Kb**	89.51	91.30	89.44	91.02	26.39
**GC contents**	38.05%	39.40%	38.33%	39.02%	40.29%
**BUSCO completeness**	98.6%	97.0%	97.0%	92.8%	98.6%
**GenBank assembly accession No.**	QLYP00000000	GCA_000004775.1	GCA_000002325.2	GCA_000004795.1	GCA_002249905.1
**Reference**	This study	(Werren *et al.* 2010)			

The 10X Genomics reads were aligned to the final assembly to compute the summary statistics. The average scaffold coverage is 671.87X and the GC-content is 41.4% (Supplemental Figure 1). RNA-seq reads from different development stages (see Methods) of *N. giraulti* were also aligned to the final assembly with an average mapping percentage of 97%, indicating a high-quality assembly of *Ng* genome. To assess the completeness of this genome, the BUSCO scores of all five genome assemblies were generated (Table 1). The BUSCO completeness score for the current assembly of *N. giraulti* is 98.6% (N = 1,066; Complete: 98.6%; Duplicated: 3.0%; Fragmented:0.4%; Missing:1.0%), indicating a high level of completeness of our genome assembly.

### Genome comparison between N. giraulti and N. vitripennis

**Figure 2:**
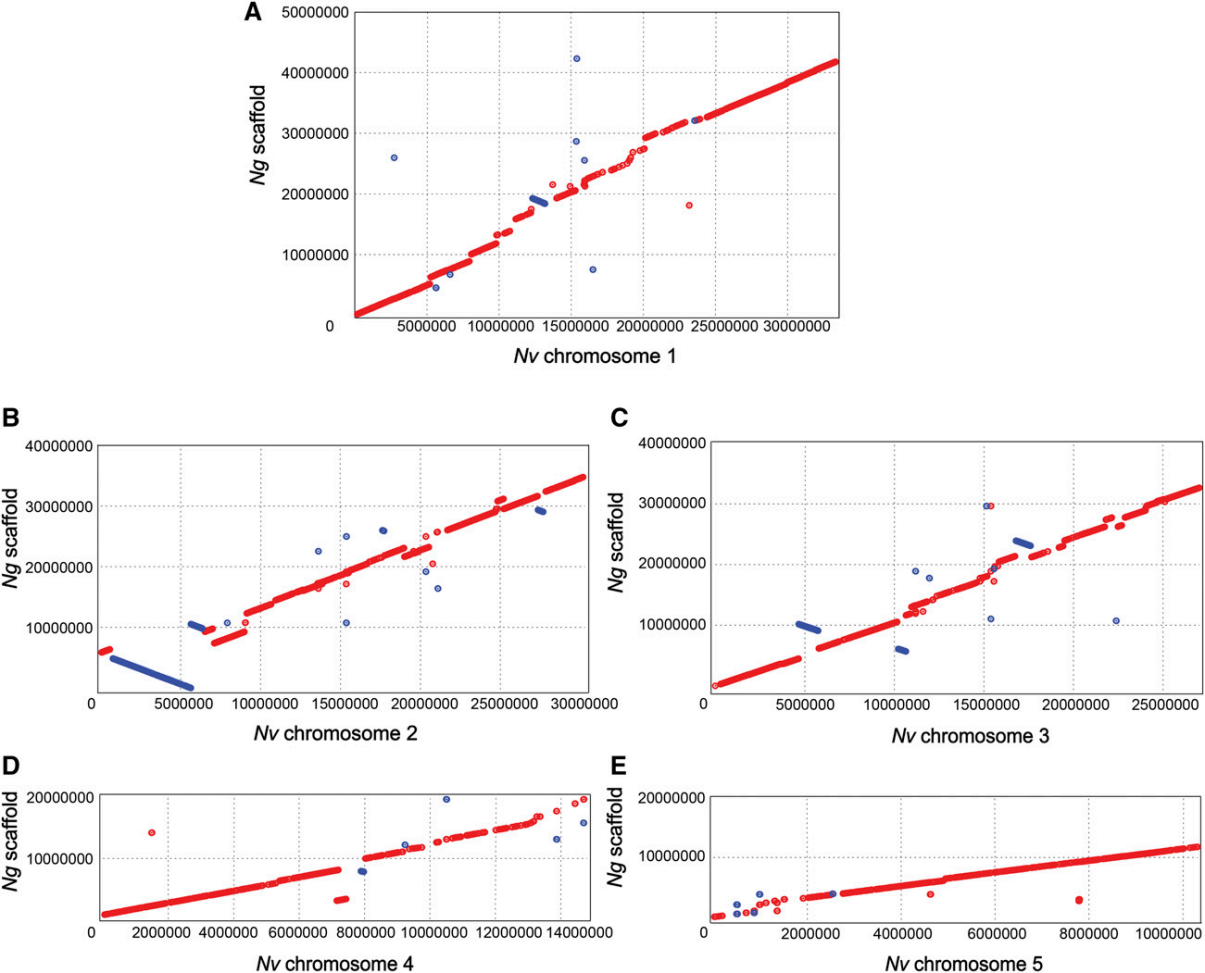
Chromosome level alignment between *N. giraulti* scaffolds and *N. vitripennis* chromosomes. Dot plot showing comparison between *N*g and *Nv* genomes. Red stands for a forward match and blue stands for a reverse match.

*Ng* scaffolds were mapped to each chromosome of the *Nv* assembly (GCA_000002325.2) (Werren *et al.* 2010) with BWA-MEM aligner (Bernt *et al.* 2013). Overall the alignments are consistent between *Ng* and *Nv* with a few insistencies (Figure 2). A total of 1,137 *Ng* scaffolds were aligned to *Nv* chromosomes (Table 2 and Supplemental Table 1), accounting for 89.3% of the total chromosome length in *Nv*. The average sequence identity in these aligned regions is 93.23%. As a useful tool for comparative analysis and interspecific mapping, we provide a set of 5,147,972 high-quality single nucleotide polymorphisms between the *Ng* and *Nv* genome assemblies (Supplemental Data 1). The SNPs fall 6.1% percent into exons (3.4% of these are synonymous and 2.7% are nonsynonymous), 16.3% percent in introns, and 77.6% percent are intragenic. These represent either species-specific or strain-specific differences, which will be resolved in the resequencing of multiple *Ng* strains in future work.

**Table 2:** Alignment length and percentage of *N. giraulti* scaffolds to *N. vitripennis* genome

*Nv* chromosome	Number of *Ng* scaffolds	Length (bp)	Sequence identity	Chromosome coverage (all)	Chromosome coverage (top 10)
**Chr1**	490	29,245,964	93.36%	87.11%	39.32%
**Chr2**	324	27,672,334	93.19%	91.34%	66.15%
**Chr3**	320	24,746,805	93.13%	91.53%	59.82%
**Chr4**	371	12,841,562	93.09%	86.72%	69.24%
**Chr5**	232	9,050,462	93.43%	87.74%	79.30%
**Total**	1,737	103,557,127	93.23%	89.30%	58.60%

### Genome annotation

In our current *N. giraulti* assembly, we have identified a total repeat content of 83,899,561 bp, by using an *Ng* specific repeat library, consisting of approximately 32.39% of the genome assembly (Table 2). Among the classified repetitive elements, the top three repeat types are DNA elements (7.58%), LINEs (6.71%) and SINEs (6.68%) (Table 3). After all the repeat regions were soft-masked by MAKER, the final annotation resulted in 14,777 protein coding genes. By comparing the annotated genes in *Ng* with *Nv*, there are 10,640 1:1 orthologs between *Ng* and *Nv*, and 83.7% *Ng* genes were assigned in orthogroups between *Ng* and *Nv*.

**Table 3:** Summary of repetitive element content found in the *N. giraulti* genome assembly

	Number of elements	Length occupied (bp)	Percentage occupied (%)
**SINEs**	586	99,652	0.04
**LINEs**	18,830	17,387,298	6.71
**LTR elements**	23,401	17,311,094	6.68
**DNA elements**	41,707	19,644,786	7.58
**Small RNA**	25	4,445	0.00
**Satellites**	1,824	745,156	0.29
**Simple repeats**	130,377	5,623,109	2.17
**Low complexity**	8,384	400,042	0.15

### Identification of genes present in Ng but not Nv

We further compared the *Ng* gene sets with the *Nv* annotated gene set OGS2 (Rago *et al.* 2016) to determine if there are any candidates for *Ng*-specific genes (see Method and Supplemental Figure S2). A total of 2,361 *Ng*-specific candidate genes were generated by Orthofinder (Emms and Kelly 2019). The protein sequences of these candidate genes were BLASTed to the *Nv* genome. A total of 112 *Ng* candidate genes showed no hits to the reference and *Nv* PSR1.1 genome assemblies (Dalla Benetta *et al.* 2020). To exclude potential pseudogenes in *Ng*, these 112 candidate genes were then aligned to the *Ng* transcripts annotated by Cufflinks (Trapnell *et al.* 2012) and 45 genes were retained. The protein sequences of these genes were aligned to *Nv* PSR1.1 again using tBLASTn and three more genes were excluded (E-value cutoff 1E-5), resulting in final list of 42 *Ng*-specific genes (Supplemental Data S2). 28 of these *Ng*-specific genes have a tBLASTn hit in *Trichomalopsis sarcophagae* (TSAR), which is a sister species to the *Nasonia* genus, suggesting that they could be degenerated genes in *Nv*. We therefore divide this class further into 28 “*Nv* absent” genes, which are not present in the annotated Nv genome but are found in the closely related species *Trichomalopsis sarcophagae*, and 14 candidate “*Ng* novel” genes, which are not found in either *Nv* or TSAR. Among these *Ng*-specific genes, eight genes are annotated with E-value < 1E-4 and identity >40% to the NCBI NR database. These include hypothetical protein TSAR_007225, NADH dehydrogenase (ubiquinone) flavoprotein 3, T-complex protein 1 subunit eta, gem associated protein 4, PREDICTED uncharacterized protein LOC107980813, collagen type II alpha, [histone H4]-N-methyl-L-lysine20 N-methyltransferase, and neuropeptides capa receptor-like gene. The BLAST2GO functional analysis revealed that these 42 genes are enriched for genes involved in gluconate transmembrane transporter activity (Supplemental Figure S3 and Data S2). The genes warrant further study to investigate their possible origins and functions.

### Phylogenomic relationship with arthropod genomes

**Figure 3:**
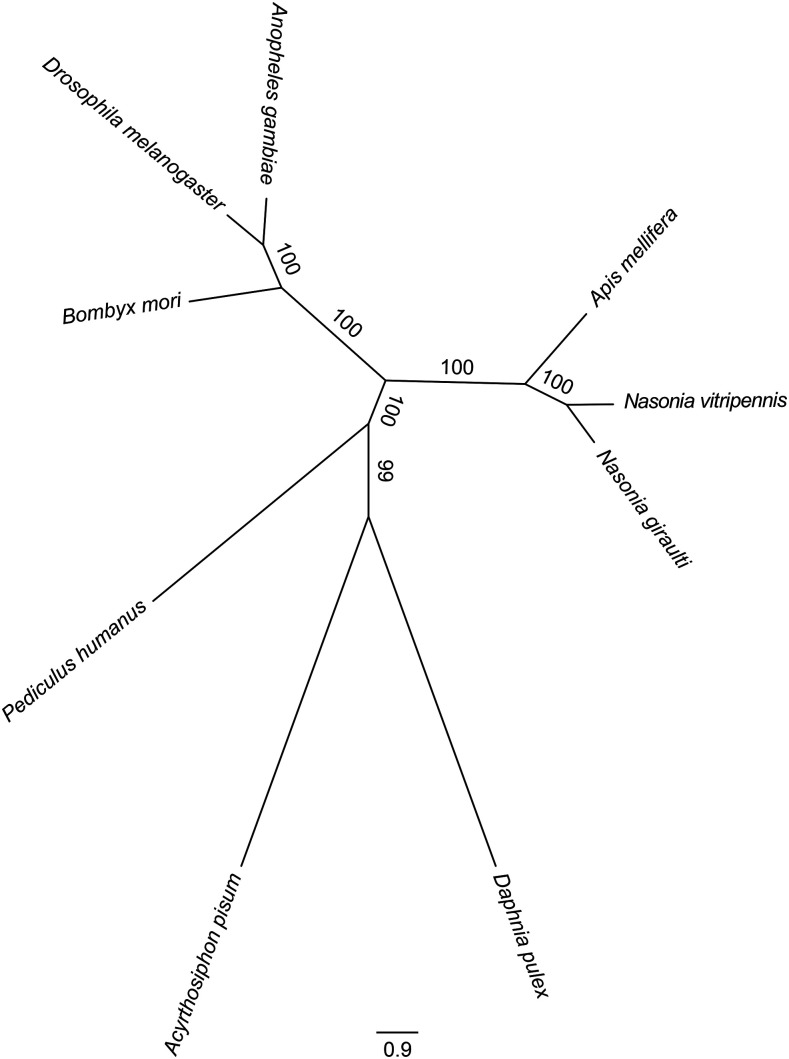
Phylogenetic relationships of *N. giraulti* with eight selected arthropod species. A phylogenetic tree of *N. giraulti* with 8 other arthropod species was constructed based on a total of 348 single-copy 1:1 orthologs. The selected arthropod genomes are from fruit fly, pea aphid, honey bee, water flea, human lice, mosquito, silk moth and jewel wasp *Nasonia vitripennis*.

We compared the *Ng* genome to 8 other sequenced arthropod genomes (fruit fly, pea aphid, honey bee, water flea, human lice, mosquito, silk moth and jewel wasp *Nv*), to identify a core gene set for phylogenomic analysis. A total of 348 single-copy 1:1 orthologs (listed in Supplemental Data S3) were identified. *Ng* is most closely related, to *Nv*, and they cluster with honey bee, another Hymenoptera species (Figure 3). These 348 single-copy ortholog provide a useful gene set for evolutionary analysis.

## Conclusions

This study describes the assembly and annotation of the genome for *Nasonia giraulti*, a key model organism in speciation and evolutionary studies that range in focus from pheromones and sex determination to behavior and memory. The assembly of 259 Mbp is very complete with a 98.6% BUSCO completeness and aligns to 89% of the genome of its sister species, *Nasonia vitripennis*. We predicted and analyzed 14,777 protein-coding genes that offer insights into the development and evolution of *N. giraulti*. We identified 5 million SNPs and 42 genes that are unique to *N. giraulti* when compared to *N. vitripennis*. This *de novo* assembled genome will provide a powerful tool in comparative genomics and evolution to the model parasitoid wasp *N. vitripennis* and will enhance future studies in the behavior, development, pheromones, repeat evolution, mitochondria-nuclear interaction, and parasitoid-host biology.
